# Retraction: Identification of Nitric Oxide as an Endogenous Inhibitor of 26S Proteasomes in Vascular Endothelial Cells

**DOI:** 10.1371/journal.pone.0342951

**Published:** 2026-02-17

**Authors:** The PLOS One Editors

Following publication of this article [1], the University of Oklahoma Health Campus (OUHC) notified PLOS that an institutional investigation determined there was falsification in Figs 1B and 11B. Specifically, OUHC reported the following findings to PLOS:

- In the Fig 1B SNP treatment panel, the same immunoblot image is used for the upper row of bands from the middle Ub-GFP panel and the β-Actin panel.
- In Fig 11B, the Poly-GFP, UB-GFP, and OGA immunoblot panels are spliced between lanes 1 and 2.

During editorial follow-up, the corresponding author JX stated that the data underlying the panels of concern are no longer available.

In light of the concerns raised about the reliability of the reported results, and in accordance with the request of the University of Oklahoma Health Campus, the *PLOS One* Editors retract this article.

JX did not agree with the retraction and stands by the article’s findings. HL, SY, and HZ either did not respond directly or could not be reached.
